# Development, Feasibility, and Acceptability of the Electronic Patient Benefit Index for Psoriasis in Clinical Practice: Mixed Methods Study

**DOI:** 10.2196/54762

**Published:** 2024-08-09

**Authors:** Marina Otten, Vahid Djamei, Matthias Augustin

**Affiliations:** 1 German Center for Health Services Research in Dermatology (CVderm) Institute for Health Services Research in Dermatology and Nursing (IVDP) University Medical Center Hamburg-Eppendorf (UKE) Hamburg Germany; 2 Department of Dermatology University Hospital Zurich Zurich Switzerland

**Keywords:** patient-reported outcomes, Patient Benefit Index, dermatology, psoriasis, feasibility, acceptability, eHealth, digital health, dermatologist, dermatologists, skin, mobile health, app, apps, application, applications, digital technology, digital intervention, digital interventions, smartphone, smartphones, mobile phone

## Abstract

**Background:**

Patient-reported outcomes are relevant in clinical practice showing patient benefits, supporting clinicians’ decision-making, and contributing to the delivery of high standards of care. Digital monitoring of patient-reported outcomes is still rare. The Patient Benefit Index (PBI) measures benefits and goals from patients’ views and may be relevant for regular documentation and shared decision-making.

**Objective:**

This study aimed to develop electronic versions of the PBI to examine their feasibility and acceptability in clinical practice for patients with psoriasis.

**Methods:**

We developed an app and a web version of the existing, valid PBI using focus groups and cognitive debriefings with patients before conducting a quantitative survey on its feasibility and acceptability. Conduction took part in an outpatient dermatology care unit in Germany. Descriptive and subgroup analyses were conducted.

**Results:**

A total of 139 patients completed the electronic PBIs (ePBIs) and took part in the survey. The ePBI was understandable (n=129-137, 92.8%-98.6%) and feasible, for example, easy to read (n=135, 97.1%) and simple to handle (n=137, 98.5%). Acceptability was also high, for example, patients can imagine using and discussing the ePBI data in practice (n=91, 65.5%) and documenting it regularly (n=88, 63.3%). They believe it could support treatment decisions (n=118, 84.9%) and improve communication with their physician (n=112, 81.3%). They can imagine filling in electronic questionnaires regularly (n=118, 84.9%), even preferring electronic over paper versions (n=113, 81.2%). Older and less educated people show less feasibility, but the latter expected the relationship with their physician to improve and would be more willing to invest time or effort.

**Conclusions:**

The app and web version of the PBI are usable and acceptable for patients offering comprehensive documentation and patient participation in practice. An implementation strategy should consider patients’ needs, barriers, and facilitators but also physicians’ attitudes and requirements from the health care system.

## Introduction

Studies already showed the relevance of patient-reported outcomes (PROs) in clinical practice [1]. PROs can provide information on the effectiveness and benefits of treatments and on changes in patient functional status over time [2] and can support patient referrals [3]. PROs can also be used to evaluate and compare health care providers and systems and can inform quality improvement efforts [2].

The National Health Service Outcomes Framework cites enhancing quality of life (QoL) for people with long-term conditions as a key domain for improvements [4]. The European Academy of Dermatology and Venereology Task Force on QoL considers that there are several ways in which the measurement of QoL in clinical practice may benefit patients, support clinicians’ decision-making, and contribute to the delivery of high standards of care [5]. PROs in dermatology include, for example, QoL, patient benefit and goal measures, treatment harm assessment, and treatment response adequacy [4,6,7]. They all enhance the management of psoriasis and, thereby, improve patients’ lives [8].

Many PROs are already included in data sets for documenting psoriasis in clinical practice [9], for example, the Patient Benefit Index (PBI) by assessing patients’ treatment goals and benefits [10]. Analyzing patient needs on an individual level facilitates shared decisions by patients and physicians and optimizes personalized treatment [11]. Furthermore, the World Health Organization [6] and the Techniker health insurance [12] emphasize the importance of patient-defined treatment goals.

The PBI (Multimedia Appendix 1) is a valid instrument suitable for the assessment of patient-reported goals and benefits in dermatological studies and practice. In the first part of the questionnaire (Patient Needs Questionnaire; PNQ), patients state how relevant 25 goals are for them on a 5-point Likert scale (0=not important at all to 4=very important). In the second part of the questionnaire (Patient Benefit Questionnaire; PBQ), they state how the identical items detect the extent to which the current therapy has contributed to attaining the therapy goals (scaled from 0=treatment did not help at all to 4=treatment helped a lot). Patients can also tick the option “does not apply to me.” The PBI total score is derived from the ratings on both questionnaires, as the PBI score is the arithmetic mean of all rated benefits (PBQ items) weighted by the relative importance of each corresponding need item (PNQ) for each patient [10,13]. The PBI has already widely been used in research.

With improved access to the internet and increased use of electronic devices, the digital health care sector gained importance. This makes the collection of electronic PROs more feasible [14,15]. Many benefits were detected by the digitalization of PROs such as error reductions, automatic scoring calculations, management of data security measures, and better access to data [16]. Furthermore, electronic PROs turned out to be feasible and acceptable to dermatologists and patient groups with different indications [3,17,18], but burdens remain [18,19]. In dermatology, digital monitoring is still rarely used [6,20] and is not evaluated in practice and for shared decision-making. The aim of this study was (1) to develop the electronic PBI (ePBI), (2) to examine its feasibility, and (3) acceptability for patients with psoriasis.

## Methods

This paper follows STROBE (Strengthening the Reporting of Observational Studies in Epidemiology) guidelines where applicable. To develop and test the ePBI, we followed 3 steps.

### Step 1: Conceptualization Phase

**Figure 1:**
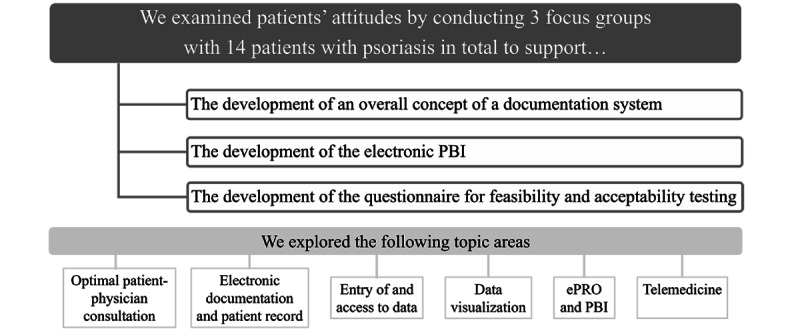
Goals and content of focus groups (besides the development of a data set for documenting psoriasis). ePRO: electronic patient-reported outcome; PBI: Patient Benefit Index.

We conducted 3 focus groups with 14 patients with psoriasis (Figure 1). The goal was to develop a basis for the conceptualization of an electronic documentation system as well as for the development of the ePBI and the questionnaire. The focus groups were recorded, fully transcribed, and subjected to content analysis [21].

### Step 2: Development of ePBI

We developed an app and a web version of the PBI [22]. For better feasibility and use in practice, adaptations were necessary:

- App: We included a first screen showing a link to PNQ and PBQ.
- App: We developed 1 page for the introduction text and each item.
- App and web: We included the calculation and presentation of resulting scores.
- App and web: We changed some words within the introduction of the PBQ and deleted or adapted the last sentence (“Please check once more if you have exactly marked each statement ...”).

Due to the PBI being a complex outcome measure and to the adaptations described earlier, we conducted further steps in developing and testing the electronic version as recommended by the Professional Society for Health Economics and Outcome Research (ISPOR) [15,23]. Cognitive debriefing allowed adaptations and iterative development of both electronic versions. Altogether, we conducted 3 rounds with 11 patients in total and used different techniques testing different parts of the ePBI until no further adjustments were necessary (Table 1). Thereby, the items of PNQ and PBQ themselves were not open for discussion.

**Table 1:** Cognitive debriefing to develop the electronic PBI^a^ versions iteratively.

Subject of investigation	PBI (app version)^b^		PBI (web version)^c^		Paper-based graphs and charts^d,e^
	Usability of PNQ^f^ and PBQ^g^	Understanding of resulting scores	Usability of PNQ and PBQ	Understanding resulting scores	Understanding and usability of paper-based graphs and charts
Technique: Think aloud	Applied	Applied	Applied	Applied	Applied
Technique: Observation	Applied	Applied	Applied	Applied	Applied
Technique: Comment options	Applied	Applied	Applied	Applied	Applied
Technique: Predefined questions^h^	Not applied	Applied	Not applied	Applied	Applied
Technique: Ad hoc questions^i^	Not applied	Applied	Not applied	Applied	Applied

^a^PBI: Patient Benefit Index.

^b^Test rounds: 2 and patient sampling: 7.

^c^Test rounds: 1 and patient sampling: 4.

^d^Test rounds: 3 and patient sampling: 11.

^e^Exemplary nondigitalized graphics were developed and discussed for future development steps.

^f^PNQ: Patient Needs Questionnaire.

^g^PBQ: Patient Benefit Questionnaire.

^h^Example: Can you describe that with your own words? Where can you find yourself on the scale?

^i^Example: When patients took a long time or showed difficulties to answer.

### Step 3: Feasibility and Acceptability Testing of ePBI

An observational study was conducted using a standardized questionnaire on patients’ demography and health status as well as on the feasibility and acceptability for both ePBI versions. We used nominal, ordinal, interval, and ratio scales. Mainly, participants answered items on 5-point Likert scales.

### Patients and Conduction

Patients were recruited at an outpatient dermatology care unit of the University Medical Center Hamburg-Eppendorf and were eligible to participate if they were German-speaking and diagnosed with psoriasis. One-half of patients filled in the app version on a smartphone. The other half filled in the web version on a laptop. After that, both groups filled in the questionnaire described earlier [15,23]. The interviewer stopped the time when each patient needed to fill out the ePBI.

### Ethical Considerations

According to the ethics commission of the Medical Association of Hamburg, there was no ethics vote required because of anonymous and noninterventional data collection for both the qualitative and quantitative parts of the study (WF-053/19). All participants gave written informed consent before participation. They were informed that they could cancel participation at any time. No compensation was provided for participation.

### Analysis

The quantitative data were analyzed using descriptive statistics. For bivariate analysis, we classified metric variables and variables with 5-point Likert scales into 3 categories. Comparison of response behaviors was conducted using cross tables, chi-square test, and Fisher exact test if cross tables had more than 20% of cells with expected counts below 5. A significance level of α=.05 was applied. For investigating the direction of the association, adjusted standardized residuals were calculated with values below –1.96 and above 1.96 revealing significance. Subgroup comparison was conducted for sex, age, education, and ePBI version (app vs web). We used SPSS (version 25.0; IBM Corp).

## Results

In total, 139 patients participated, and all filled in the questionnaire on demography, feasibility, and acceptability: 52.5% (n=73) completed the app, and 47.5% (n=66) completed the web version.

### Participants

The sample encompassed 67.6% (n=94) of male participants and had a mean age of 47 (SD 13.95; median 49; range 18-84) years. On average, the first psoriasis symptoms appeared 22 (SD 14.66; median 19; range 0-65) years ago, and the first diagnoses had been made 21 (SD 15.15; median 19; range 0-65) years ago. The mean Dermatology Life Quality Index score was 4.57 (SD 5.50; median 3; range 0-22), the mean EQ-5D-5L score was 83.05 (SD 21.92; median 88; range 8.83-100), the mean European Quality Visual Analogue Scale score was 73.93 (SD 20.43; median 80; range 1-100), and the mean PBI score 2.77 (SD 1.13; median 3.11; range 0.10-4.0). Most participants were of higher education (intermediate and high: n=107, 76.9%), and had several comorbidities (eg, n=58, 41.7% had psoriasis arthritis; on average, a patient had 0.98 comorbidities; Tables 2 and 3).

The majority was experienced in using internet-ready devices (eg, n=124, 89.9% used smartphones frequently; Table 4).

**Table 2:** Descriptive results of demographic and clinical information I (N=139).

	Mean (SD)	Median	Mode	Minimum	Maximum
Age (n=139)	47 (13.95)	49	50	18	84
First symptoms appeared/years ago (n=132)	22 (14.66)	19	20	0	65
Diagnosis made/years ago (n=130)	21 (15.15)	19	10	0	65
Number of comorbidities (n=135)	0.98 (0.99)	1	1	0	5
Dermatology Life Quality Index; range 0-30 (n=135)	4.57 (5.50)	3	0	0	22
EQ-5D-5L; range 0-100 (n=134)	83.05 (21.92)	88	100	8.83	100
EQ VAS^a^; range 0-100 (n=125)	73.93 (20.43)	80	90	1	100
Electronic Patient Benefit Index; range 0-100 (n=89^b^)	2.77 (1.13)	3.11	4	0.1	4

^a^EQ VAS: European Quality Visual Analogue Scale.

^b^Patient Benefit Index global score and subscales may only be computed if the patient has provided valid data on importance (Patient Needs Questionnaire) and benefit (Patient Benefit Questionnaire) for at least 75% of the respective treatment goals.

**Table 3:** Descriptive results of demographic and clinical information II (N=139).

		Values, n (%)
**Sex**		
	Male	94 (67.6)
	Female	44 (31.7)
	Missing	1 (0.7)
**Highest school diploma**		
	No degree	2 (1.4)
	Lower or general secondary degree	23 (16.5)
	Intermediate secondary degree	42 (30.2)
	Polytechnic high school degree	5 (3.6)
	University of applied sciences entrance qualification	22 (15.8)
	Higher education entrance qualification	38 (27.3)
	Missing	3 (2.2)
	Other degree	4 (2.9)
**Comorbidities**		
	Psoriasis arthritis	58 (41.7)
	Diseases of the cardiovascular system (eg, high blood pressure and arteriosclerosis)	21 (15.2)
	Diabetes mellitus type 2	6 (4.3)
	Obesity	15 (10.8)
	Lipometabolic disorders	3 (2.2)
	Depression	18 (12.9)
	Nonalcoholic steatohepatitis (fatty liver disease)	4 (2.9)
	Chronic inflammatory bowel diseases (eg, Morbus Crohn)	1 (0.7)
	Alcohol abuse	0 (0)
	Nicotine abuse	7 (5)
	None	48 (34.5)

**Table 4:** Descriptive results of the use of internet-ready devices (N=139).

	Computer, n (%)	Laptop, n (%)	Tablet-PC, n (%)	Smartphone, n (%)
Not at all	39 (28.3)	30 (21.7)	44 (31.9)	11 (8)
Rarer than 1 time a week	11 (8)	21 (15.2)	16 (11.6)	1 (0.7)
1-3 times a week	9 (6.5)	15 (10.9)	12 (8.7)	0 (0)
4-6 times a week	3 (2.2)	14 (10.1)	5 (3.6)	3 (2.2)
Daily	63 (45.7)	44 (31.9)	44 (31.9)	120 (87)
Missing	13 (9.4)	14 (10.1)	17 (12.3)	3 (2.2)

### Feasibility

Most participants (“rather” or “totally”) agreed on the ePBI being usable (Table 5), stating that the questions and texts were easy to read (n=135, 97.1%), the questions (n=133, 95.7%) or instructions (n=137, 98.6%) or information (n=129, 92.8%) understandable, the handling simple (n=137, 98.5%), the questionnaire easy to complete (n=136, 97.8%), and the information on individual results satisfying (n=122, 87.8%). A small number of participants declared that they needed help filling in the ePBI (n=24, 17.3%) and found it too long (n=30, 21.6%) but visually appealing (n=117, 84.2%; Table 3). On average, they needed 6.92 (SD 2.49; median 7; range 2.75-14) minutes to fill in the electronic PNQ and PBQ, read the results, and the last page including the PBI score (Table 5).

**Table 5:** Descriptive results on usability (N=139)^a^.

	Totally disagree, n (%)	Rather not agree, n (%)	Partly agree, n (%)	Rather agree, n (%)	Totally agree, n (%)	Missing, n (%)
The questions and texts are easy to read.	0 (0)	1 (0.7)	2 (1.4)	10 (7.2)	125 (89.9)	1 (0.7)
The questions are understandable.	0 (0)	1 (0.7)	5 (3.6)	14 (10.1)	119 (85.6)	0 (0)
The handling is simple.	0 (0)	0 (0)	1 (0.7)	12 (8.6)	125 (89.9)	1 (0.7)
The instructions on how to complete the questionnaire are understandable.	0 (0)	0 (0)	1 (0.7)	16 (11.5)	121 (87.1)	1 (0.7)
In general, the questionnaire is easy to complete.	0 (0)	1 (0.7)	2 (1.4)	12 (8.6)	124 (89.2)	0 (0)
The information about my individual results is understandable.	0 (0)	1 (0.7)	6 (4.3)	35 (25.2)	94 (67.6)	3 (2.2)
I am satisfied with the content of the information about my individual results.	0 (0)	5 (3.6)	9 (6.5)	37 (26.6)	85 (61.2)	3 (2.2)
I need help filling in the questionnaires.	77 (55.4)	27 (19.4)	9 (6.5)	5 (3.6)	19 (13.7)	2 (1.4)
The questionnaire seems too long to me.	42 (30.2)	51 (36.7)	16 (11.5)	19 (13.7)	11 (7.9)	0 (0)
The questionnaire is visually appealing.	0 (0)	4 (2.9)	17 (12.2)	56 (40.3)	61 (43.9)	1 (0.7)

^a^Completion time (n=138): mean 6.92, SD 2.49; median 7; mode 6; range 2.75-14 minutes.

### Acceptability

Acceptability of the ePBI was also very high (Tables 6-9) but with more participants being indecisive (between n=7, 5% and n=41, 29.5%). Most participants (“rather” or “totally agreed”) could imagine filling in the ePBI on a regular basis (n=88, 63.3%), but less so if the documentation would be only for their own use (n=55, 39.5% and n=34, 24.5% being indecisive). They could imagine to discuss the content with their physician (n=91, 65.5%). They thought the ePBI could form a good basis for a patient-physician consultation (n=98, 70.5%), support treatment decisions (n=118, 84.9%), improve communication with the physician (n=113, 81.3%), and improve the relationship with the physician (n=78, 56.1%). In addition, it would help them to remember their symptoms and well-being better during patient-physician consultation (n=94, 67.7%). It would also help them to manage their condition (n=90, 64.7%; over periods of time: n=129, 92.8%). In general, patients could imagine filling in electronic questionnaires (n=118, 84.9%). They did not agree that regular completion might make them feel sad (n=120, 86.3%) and they would not prefer filling in the questionnaires on paper (n=113, 81.2%; Table 6). The majority of participants could imagine filling in the ePBI at every patient-physician consultation (n=85, 61.2%; Table 7). However, they accepted much greater effort for documenting their data regarding frequency, length, and device (Tables 7-9).

**Table 6:** Descriptive results on acceptability I (N=139).

	Totally disagree, n (%)	Rather not agree, n (%)	Partly agree, n (%)	Rather agree, n (%)	Totally agree, n (%)	Missing, n (%)
I can imagine filling in the questionnaire regularly.	5 (3.6)	16 (11.5)	29 (20.9)	42 (30.2)	46 (33.1)	1 (0.7)
The regular answering of the questions would make me feel sad.	84 (60.4)	36 (25.9)	10 (7.2)	2 (1.4)	7 (5)	0 (0)
I would rather answer the questions on paper.	78 (56.1)	35 (25.1)	12 (8.6)	6 (4.3)	7 (5)	1 (0.7)
I can imagine discussing my personal goals and benefits from the questionnaires with my physician.	10 (7.2)	7 (5)	31 (22.3)	47 (33.8)	44 (31.7)	0 (0)
The questionnaires on my personal goals and benefits form a good basis for a patient-physician consultation.	3 (2.2)	9 (6.5)	28 (20.1)	46 (33.1)	52 (37.4)	1 (0.7)
I can imagine filling out and documenting the questionnaires for myself.	20 (14.4)	29 (20.9)	34 (24.5)	23 (16.5)	32 (23)	1 (0.7)
I can basically imagine filling out electronic questionnaires.	5 (3.6)	4 (2.9)	11 (7.9)	35 (25.2)	83 (59.7)	1 (0.7)
By filling out and storing the data on my goals and benefits, treatment decisions can be supported.	0 (0)	1 (0.7)	20 (14.4)	51 (36.7)	67 (48.2)	0 (0)
By filling out and storing the data on my goals and benefits, communication with my physician can be improved.	0 (0)	5 (3.6)	21 (15.1)	38 (27.3)	75 (54)	0 (0)
By filling in and storing the data on my goals and benefits, the relationship with my physician can be improved.	4 (2.9)	16 (11.5)	41 (29.5)	28 (20.1)	50 (36)	0 (0)
By filling in and saving the data on my goals and benefits, the course of my disease can be observed over a long period of time.	1 (0.7)	2 (1.4)	7 (5)	42 (30.2)	87 (62.6)	0 (0)
By filling in and saving the data on my goals and benefits, I can better remember my symptoms and well-being during patient-physician consultation.	6 (4.3)	12 (8.6)	27 (19.4)	34 (24.5)	60 (43.2)	0 (0)
By filling in and storing the data on my goals and benefits, I can gain more control over my condition.	4 (2.9)	14 (10.1)	31 (22.3)	38 (27.3)	52 (37.4)	0 (0)

**Table 7:** Descriptive results on acceptability II (N=139).

		Values, n (%)
**How often would you be willing to fill in the questionnaires on your personal goals and benefits?**		
	Not at all	1 (0.7)
	Rarer than at each patient-physician consultation	33 (23.7)
	At every patient-physician consultation	85 (61.2)
	More frequently than at each patient-physician consultation	14 (10.1)
	Missing	6 (4.3)

**Table 8:** Descriptive results on acceptability III (N=139).

			Daily, n (%)		Weekly, n (%)	Monthly, n (%)
**What is the maximum amount of minutes it may take, that you would fill in the data ...**						
	That would be too often for me	79 (56.8)		31 (22.3)		1 (0.7)
	1 minute	10 (7.2)		6 (4.3)		2 (1.4)
	5 minutes	29 (20.9)		50 (36)		27 (19.4)
	10 minutes	15 (10.8)		35 (25.2)		69 (49.6)
	20 minutes	0 (0)		9 (6.5)		22 (15.8)
	30 minutes	1 (0.7)		2 (1.4)		11 (7.9)
	Over 30 minutes	1 (0.7)		1 (0.7)		4 (2.9)
	Missing	4 (2.9)		5 (3.6)		3 (2.2)

**Table 9:** Descriptive results on acceptability IV (N=139).

		From home, n (%)	In the waiting room, n (%)	Via my own smartphone or tablet, n (%)	Via my own laptop or PC, n (%)	Via a device provided to me by the physician (eg, smartphone, tablet, and laptop), n (%)
**How could you imagine filling in the questionnaires about your personal goals and benefits?**						
	Totally disagree	16 (11.5)	3 (2.2)	23 (16.5)	31 (22.3)	23 (16.5)
	Rather not agree	7 (5)	14 (10.1)	19 (13)	20 (14.4)	13 (9.4)
	Neither	13 (9.4)	26 (18.7)	12 (8.7)	9 (6.5)	14 (10.1)
	Rather agree	16 (11.5)	23 (16.5)	17 (13.7)	15 (10.8)	18 (12.9)
	Totally agree	87 (62.6)	71 (51.1)	68 (48.9)	60 (43.2)	70 (50.4)
	Missing	0 (0)	2 (1.4)	0 (0)	4 (2.9)	1 (0.7)

### Subgroup Comparison

Subgroup comparison showed some significant differences regarding sex, age, education, and ePBI version (web or app; Multimedia Appendix 2). Female participants disagreed more often than male participants that data collection of ePBI can improve communication between physician and patient. They were more often of the opinion that they could gain more control over their disease and showed significantly more missing values on the question if they could imagine filling in the ePBI via their own laptop or PC (see adjusted residuals in Multimedia Appendix 2).

Patients older than 60 years were less likely to think that filling in e-surveys daily was too frequent. They were willing to spend more time on completing (long) questionnaires although it took them more time. The younger group even needed less assistance. Anyway, the older groups still preferred completion on paper, respectively, less often to fill in electronic questionnaires in comparison to the younger groups.

Patients with low or no school degrees expected the relationship with their physician to improve by filling in the ePBI. They were less willing to spend a lot of time on questionnaire completion than the higher education groups, as it also took them longer to complete an ePBI.

Only one significant difference was found between the opinions of patients filling in the app in comparison to the web version: patients who filled in the web version more often thought that the questionnaire would form a good basis for a patient-physician consultation than the patients who used the app (see adjusted residuals in Multimedia Appendix 2).

## Discussion

### Summary

We developed an app and a web version of the existing and valid PBI using the results of focus groups for conceptualization and cognitive debriefing to adapt the preliminary versions as recommended by the ISPOR. The results of the subsequent survey showed a high understanding and feasibility of the ePBI. Moreover, the acceptability was also very high: patients can imagine using and discussing the ePBI in practice and documenting it regularly, expecting improvements, for example, better treatment decisions and communication with their physician, as well as gaining more control over their disease. Importantly, they can imagine filling in the ePBI and other electronic questionnaires regularly, even preferring the electronic over the paper versions. Other studies also showed high feasibility and acceptability of electronic data collection [19,24,25]. These results show that the basis for an implementation is in place. However, the acceptability of patients with dermatological diseases is higher compared to dermatologists’ acceptability [19,24], indicating barriers for implementation as physicians play a key role in disseminating [26].

### Principal Findings

The frequent values of the “partly agree” answers (Likert scale) to the acceptability questions show that some patients may have difficulties in imagining the use of an electronic PRO in clinical practice. We assume that the more experiences people gain with electronic devices, the more usable and acceptable it will become.

We did not find fundamental differences in sex. Merely, female participants in our study would use the ePBI more often than male participants to gain more control over their disease, which goes in line with previous findings showing that female participants are more health-conscious than male participants [27]. According to male participants, the ePBI could help to improve communication with their physician, which may be more important for them to improve than for female participants.

We detected several differences in age, which are reinforced by other studies, after which older adults use technologies at lower rates than other age groups [28]. In this study, the older participants had more problems filling in the ePBI and would more often prefer paper versions over digital ones. This may indicate a digital divide. However, they were willing to spend more time documenting data digitally, probably reflecting their higher needs. Existing data also reveal that internet use by older people is increasing, expecting a less digital divide in the future [28].

Participants with lower school degrees had more difficulties filling in the ePBI and were less willing to spend time documenting their data than those with higher education. We assume that it takes them longer to understand the concept of and get used to such digital applications. Again, this may indicate a digital divide. Nevertheless, our data reveal that it may be especially promising for this group to use the ePBI, as participants with lower school degrees more often expected an improved relationship with their physicians.

We only found one difference between the participants’ opinions using the different modes of data collection. They thought the web version would be more suitable to form a good basis for a patient-physician consultation than the app. In the web version, the questionnaire looks more familiar, as the structure is similar to a paper version. It is also easier to discuss data on a big screen than on a mobile phone. However, as soon as data are digitalized, there are several opportunities to display and store them on different electronic devices. Again, we assume that gaining more experience in data collection and use will increase usability and acceptability.

Due to its many years of proven use in research and practice, we have conducted various qualitative studies on the development of the ePBI. We followed guidelines on research, implementation, and validation processes. The sampling size was chosen based on statistical necessity and therefore appropriate to answer the present research question. As recruitment was conducted in a specified outpatient ward, the sample mainly includes patients who are moderately and severely affected. Digital applications especially aim to support these patient groups, as they have more issues dealing with their disease. Our sample consists of many patients who are well-educated and experienced regarding internet-ready devices, possibly resulting in more positive attitudes on feasibility and acceptability as our results show with respect to the subgroup analysis. The digitalization rate is only slightly higher than within the general population, for example, in Germany [29]. As nearly everybody within our sample had great experiences with digital devices, we did not expect significant differences between low or high users within our sample. Unfortunately, we did not capture the number of patients who were asked to participate but declined. Therefore, we have no information about that group.

### Conclusions

The app and web version of the ePBI are usable and acceptable for patients. The results show that the basis for an implementation is in place. This offers many advantages not only for research but also for documenting and more comprehensively using patient data in practice, such as in eHealth records or other documentation applications, for shared decision-making and patient participation. Thereby, a focus should be placed on the digital divide with special attention on age and education. People with higher age and lower education seem to have more difficulties using and understanding digital applications. In contrast, they are willing to put more effort into documentation and see other important advantages, which may increase successful use. Information on and experiences with digital applications, such as the ePBI or other digital monitoring solutions, will increase usability and acceptability.

Our results can support other researchers, developers, and decision makers. Physicians need to be better informed and included in processes, as their low acceptability and use may form a barrier for future implementations. In Germany, some political barriers also hinder digitalization in medicine, for example, no unified telematics infrastructure, reimbursement, and data security issues.

## Appendix

Multimedia Appendix 1 Patient Benefit Index questionnaire.

Multimedia Appendix 2 Significant results of subgroup analysis.

## Abbreviations

ePBI: electronic Patient Benefit Index
ISPOR: Professional Society for Health Economics and Outcome Research
PBI: Patient Benefit Index
PBQ: Patient Benefit Questionnaire
PNQ: Patient Needs Questionnaire
PRO: patient-reported outcome
QoL: quality of life
STROBE: Strengthening the Reporting of Observational Studies in Epidemiology

## Data Availability

The data sets generated and analyzed during this study are available from the corresponding author on reasonable request.
