# Supplementary material for: Development, Feasibility, and Acceptability of the Electronic Patient Benefit Index for Psoriasis in Clinical Practice: Mixed Methods Study
Source: JMIR Dermatol. 2024 Aug 9;7:e54762. doi: 10.2196/54762 (PMC11344180; doi:10.2196/54762)
Supplement: Multimedia Appendix 1 [file derma_v7i1e54762_app1.pdf]

Importance of Treatment Goals

With the help of the following questions, we'd like to know how important the goals mentioned below are to you personally in the **current treatment** of your skin disease.

For each of the following statements, please mark **how important** this treatment goal is to you. If a statement does not apply to you, for example because you are not experiencing pain, please mark "*does not apply to me*".

	**As a result of therapy, how important is it for you to ...**	**not at all**	**somewhat**	**moderately**	**quite**	**very**	*does not apply to me*
1	...be free of pain	O	O	O	O	O	O
2	...be free of itching	O	O	O	O	O	O
3	...no longer have burning sensations on your skin	O	O	O	O	O	O
4	...be healed of all skin defects	O	O	O	O	O	O
5	...sleep better	O	O	O	O	O	O
6	...feel less depressed	O	O	O	O	O	O
7	...experience greater enjoyment of life	O	O	O	O	O	O
8	...have no fear that the disease will get worse	O	O	O	O	O	O
9	...lead a normal everyday life	O	O	O	O	O	O
10	...be more productive in everyday life	O	O	O	O	O	O
11	...be less of a burden to relatives and friends	O	O	O	O	O	O
12	...engage in normal leisure activities	O	O	O	O	O	O
13	...be able to lead a normal working life	O	O	O	O	O	O
14	...be able to have more contact with other people	O	O	O	O	O	O
15	…be more comfortable showing yourself in public	O	O	O	O	O	O
16	...be less burdened in your partnership	O	O	O	O	O	O
17	...be able to have a normal sex life	O	O	O	O	O	O
18	...be less dependent on doctor and clinic visits	O	O	O	O	O	O
19	...need less time for daily treatment	O	O	O	O	O	O
20	...have fewer out-of-pocket treatment expenses	O	O	O	O	O	O
21	...have fewer side effects	O	O	O	O	O	O
22	...find a clear diagnosis and therapy	O	O	O	O	O	O
23	...have confidence in the therapy	O	O	O	O	O	O
24	...get better skin quickly	O	O	O	O	O	O
25	...regain control of the disease	O	O	O	O	O	O

Please recheck your answers to make sure your have clearly marked each statement with an "x".

Thank you very much for your cooperation!

Treatment Benefits

When the treatment began, you indicated in a questionnaire how important various goals were in the treatment of your skin disease.

Please mark each of the following statements according to the extent to which those treatment goals **were achieved**, indicating if the treatment has benefited you. If a statement did not apply to you, for example because you did not experience any pain, please mark "*did not apply to me*".

	**The current treatment has helped me to …**	**not at all**	**somewhat**	**moderately**	**quite**	**very**	*did not apply to me*
1	...be free of pain	O	O	O	O	O	O
2	...be free of itching	O	O	O	O	O	O
3	...no longer have burning sensations on my skin	O	O	O	O	O	O
4	...be healed of all skin defects	O	O	O	O	O	O
5	...sleep better	O	O	O	O	O	O
6	...feel less depressed	O	O	O	O	O	O
7	...experience greater enjoyment of life	O	O	O	O	O	O
8	...have no fear that the disease will get worse	O	O	O	O	O	O
9	...lead a normal everyday life	O	O	O	O	O	O
10	...be more productive in everyday life	O	O	O	O	O	O
11	...be less of a burden to relatives and friends	O	O	O	O	O	O
12	...engage in normal leisure activities	O	O	O	O	O	O
13	...be able to lead a normal working life	O	O	O	O	O	O
14	...be able to have more contact with other people	O	O	O	O	O	O
15	…be more comfortable showing myself in public	O	O	O	O	O	O
16	...be less burdened in my partnership	O	O	O	O	O	O
17	...be able to have a normal sex life	O	O	O	O	O	O
18	...be less dependent on doctor and clinic visits	O	O	O	O	O	O
19	...need less time for daily treatment	O	O	O	O	O	O
20	...have fewer out-of-pocket treatment expenses	O	O	O	O	O	O
21	...have fewer side effects	O	O	O	O	O	O
22	...find a clear diagnosis and therapy	O	O	O	O	O	O
23	...have confidence in the therapy	O	O	O	O	O	O
24	...get better skin quickly	O	O	O	O	O	O
25	...regain control of the disease	O	O	O	O	O	O

Please recheck your answers to make sure your have clearly marked each statement with an "x".

Thank you very much for your cooperation!
