# Supplementary material for: Development, Feasibility, and Acceptability of the Electronic Patient Benefit Index for Psoriasis in Clinical Practice: Mixed Methods Study
Source: JMIR Dermatol. 2024 Aug 9;7:e54762. doi: 10.2196/54762 (PMC11344180; doi:10.2196/54762)
Supplement: Multimedia Appendix 2 [file derma_v7i1e54762_app2.docx]

| **Gender (N = 139)** | | | | | | | | | | | | | | | | | | | | | | | | | | | | |
| --- | --- | --- | --- | --- | --- | --- | --- | --- | --- | --- | --- | --- | --- | --- | --- | --- | --- | --- | --- | --- | --- | --- | --- | --- | --- | --- | --- | --- |
| **By filling out and storing the data on my goals and benefits, communication with my physician can be improved. (p = 0.035)** | | | | | | | | | | | | | | | | | | | | | | | | | | | | |
|  | | | | Male | | | | | | | | | | | | | Female | | | | | | | | | | | |
|  | | | | N (%) | | | | | | | Adjusted residual | | | | | | N (%) | | | | | | | Adjusted residual | | | | |
| Disagree | | | | 1 (1.1%) | | | | | | | **-2.4** | | | | | | 4 (9.1%) | | | | | | | **2.4** | | | | |
| Neither agree nor disagree | | | | 17 (18.1%) | | | | | | | 1.4 | | | | | | 4 (9.1%) | | | | | | | -1.4 | | | | |
| Agree | | | | 76 (80.9%) | | | | | | | -0.1 | | | | | | 36 (81.8%) | | | | | | | 0.1 | | | | |
| Missing | | | | 0 (0%) | | | | | | | 0 (0%) | | | | | | 0 (0%) | | | | | | | 0 (0%) | | | | |
| **By filling in and storing the data on my goals and benefits, I can gain more control over my condition. (p = 0.044)** | | | | | | | | | | | | | | | | | | | | | | | | | | | | |
|  | | | | Male | | | | | | | | | | | | | Female | | | | | | | | | | | |
|  | | | | N (%) | | | | | | | Adjusted residual | | | | | | N (%) | | | | | | | Adjusted residual | | | | |
| Disagree | | | | 15 (16.0%) | | | | | | | 1.5 | | | | | | 3 (6.8%) | | | | | | | -1.5 | | | | |
| Neither agree nor disagree | | | | 25 (26.6%) | | | | | | | 1.7 | | | | | | 6 (13.6%) | | | | | | | -1.7 | | | | |
| Agree | | | | 54 (57.4%) | | | | | | | **-2.5** | | | | | | 35 (79.5%) | | | | | | | **2.5** | | | | |
| Missing | | | | 0 (0%) | | | | | | | 0 (0%) | | | | | | 0 (0%) | | | | | | | 0 (0%) | | | | |
| **Can you imagine filling in the electronic questionnaires about your personal goals and benefits using your laptop/PC? (p = 0.025)** | | | | | | | | | | | | | | | | | | | | | | | | | | | | |
|  | | | | Male | | | | | | | | | | | | | Female | | | | | | | | | | | |
|  | | | | N (%) | | | | | | | Adjusted residual | | | | | | N (%) | | | | | | | Adjusted residual | | | | |
| Disagree | | | | 37 (39.4%) | | | | | | | 0.9 | | | | | | 14 (31.8%) | | | | | | | -0.9 | | | | |
| Neither agree nor disagree | | | | 5 (5.3%) | | | | | | | -0.8 | | | | | | 4 (9.1%) | | | | | | | 0.8 | | | | |
| Agree | | | | 52 (55.3%) | | | | | | | 0.6 | | | | | | 22 (50.0%) | | | | | | | -0.6 | | | | |
| Missing | | | | 0 (0%) | | | | | | | **-3.0** | | | | | | 4 (9.1%) | | | | | | | **3.0** | | | | |
| **Age (N = 139)** | | | | | | | | | | | | | | | | | | | | | | | | | | | | |
| **What is the maximum amount of minutes it may take, that you would fill in the data daily? (p = 0.014)** | | | | | | | | | | | | | | | | | | | | | | | | | | | | |
|  | | | | 18–39 years old | | | | | | | | 40–59 years old | | | | | | | | | 60+ years old | | | | | | | |
|  | | | | N (%) | | | | Adjusted residual | | | | N (%) | | | | Adjusted residual | | | | | N (%) | | | | Adjusted residual | | | |
| That would be too often for me | | | | 26 (60.5%) | | | | 0.6 | | | | 44 (64.7%) | | | | 1.8 | | | | | 9 (32.1%) | | | | **-3.0** | | | |
| 1–5 minutes | | | | 13 (30.2%) | | | | 0.4 | | | | 18 (26.5%) | | | | -0.4 | | | | | 8 (28.6%) | | | | 0.1 | | | |
| 10–20 minutes | | | | 3 (7.0%) | | | | -1.0 | | | | 4 (5.9%) | | | | 0 | | | | | 8 (28.6%) | | | | 1.1 | | | |
| 30+ minutes | | | | 0 (0%) | | | | -1.0 | | | | 1 (1.5%) | | | | 0 | | | | | 1 (3.6%) | | | | 1,1 | | | |
| Missing | | | | 1 (2.3%) | | | | -0.3 | | | | 1 (1.5%) | | | | -1,0 | | | | | 2 (7.1%) | | | | 1.5 | | | |
| **What is the maximum amount of minutes it may take, that you would fill in the data weekly? (p = 0.025)** | | | | | | | | | | | | | | | | | | | | | | | | | | | | |
|  | | | | 18–39 years old | | | | | | | | 40–59 years old | | | | | | | | | 60+ years old | | | | | | | |
|  | | | | N (%) | | | | Adjusted residual | | | | N (%) | | | | Adjusted residual | | | | | N (%) | | | | Adjusted residual | | | |
| That would be too often for me | | | | 9 (20.9%) | | | | -0.3 | | | | 17 (25.0%) | | | | 0.7 | | | | | 5 (17.9%) | | | | -0.6 | | | |
| 1–5 minutes | | | | 24 (55.8%) | | | | **2.5** | | | | 26 (38.2%) | | | | -0.5 | | | | | 6 (21.4%) | | | | **-2.3** | | | |
| 10–20 minutes | | | | 9 (20.9%) | | | | -1.8 | | | | 23 (33.8%) | | | | 0.5 | | | | | 12 (42.9%) | | | | 1.4 | | | |
| 30+ minutes | | | | 0 (0%) | | | | -1.2 | | | | 1 (1.5%) | | | | -0.5 | | | | | 2 (7.1%) | | | | **2.0** | | | |
| Missing | | | | 1 (2.3%) | | | | -0.5 | | | | 1 (1.5%) | | | | -1.3 | | | | | 3 (10.7%) | | | | **2.3** | | | |
| **Can you imagine filling out the electronic questionnaires about your personal goals and benefits using your smartphone/tablet? (p = 0.014)** | | | | | | | | | | | | | | | | | | | | | | | | | | | | |
|  | | | | 18–39 years old | | | | | | | | 40–59 years old | | | | | | | | | 60+ years old | | | | | | | |
|  | | | | N (%) | | | | Adjusted residual | | | | N (%) | | | | Adjusted residual | | | | | N (%) | | | | Adjusted residual | | | |
| Disagree | | | | 7 (16.3%) | | | | **-2,4** | | | | 20 (29.4%) | | | | -0.2 | | | | | 15 (53.6%) | | | | **3.0** | | | |
| Neither agree nor disagree | | | | 3 (7.0%) | | | | -0.5 | | | | 8 (11.8%) | | | | 1.3 | | | | | 1 (3.6%) | | | | -1.1 | | | |
| Agree | | | | 33 (76.7%) | | | | **2.5** | | | | 40 (58.8%) | | | | -0.6 | | | | | 12 (42.9%) | | | | **-2.2** | | | |
| Missing | | | | 0 (0%) | | | | 0 (0%) | | | | 0 (0%) | | | | 0 (0%) | | | | | 0 (0%) | | | | 0 (0%) | | | |
| **Can you imagine filling out the electronic questionnaires about your personal goals and benefits using a device (eg, smartphone, tablet, laptop) provided at the doctor’s office? (p = 0.006)** | | | | | | | | | | | | | | | | | | | | | | | | | | | | |
|  | | | | 18–39 years old | | | | | | | | 40–59 years old | | | | | | | | | 60+ years old | | | | | | | |
|  | | | | N (%) | | | | Adjusted residual | | | | N (%) | | | | Adjusted residual | | | | | N (%) | | | | Adjusted residual | | | |
| Disagree | | | | 6 (14.0%) | | | | **-2.2** | | | | 16 (23.5%) | | | | -0.6 | | | | | 14 (50,0%) | | | | **3.3** | | | |
| Neither agree nor disagree | | | | 2 (4.7%) | | | | -1.4 | | | | 10 (14.7%) | | | | 1.8 | | | | | 2 (7.15%) | | | | -0.6 | | | |
| Agree | | | | 35 (81.4%) | | | | **3.1** | | | | 41 (60.3%) | | | | -0.7 | | | | | 12 (42.9%) | | | | **-2.5** | | | |
| Missing | | | | 0 (0%) | | | | -0.7 | | | | 1 (1.5%) | | | | 1.0 | | | | | 0 (0%) | | | | -0.5 | | | |
| **I need assistance in filling out the questionnaires. (p = 0.001)** | | | | | | | | | | | | | | | | | | | | | | | | | | | | |
|  | | | | 18–39 years old | | | | | | | | 40–59 years old | | | | | | | | | 60+ years old | | | | | | | |
|  | | | | N (%) | | | | Adjusted residual | | | | N (%) | | | | Adjusted residual | | | | | N (%) | | | | Adjusted residual | | | |
| Disagree | | | | 36 (83.7%) | | | | 1.6 | | | | 54 (79.4%) | | | | 1.2 | | | | | 14 (50.0%) | | | | **-3.4** | | | |
| Neither agree nor disagree | | | | 4 (9.3%) | | | | 0.9 | | | | 1 (1.5%) | | | | **-2.3** | | | | | 4 (14.3%) | | | | 1.9 | | | |
| Agree | | | | 3 (7.0%) | | | | **-2.1** | | | | 13 (19.2%) | | | | 0.6 | | | | | 8 (28.6%) | | | | 1.8 | | | |
| Missing | | | | 0 (0%) | | | | -1.0 | | | | 0 (0%) | | | | -1.4 | | | | | 2 (7.1%) | | | | **2.8** | | | |
| **The questionnaire seems too long to me. (p = 0.037)** | | | | | | | | | | | | | | | | | | | | | | | | | | | | |
|  | | | | 18–39 years old | | | | | | | | 40–59 years old | | | | | | | | | 60+ years old | | | | | | | |
|  | | | | N (%) | | | | Adjusted residual | | | | N (%) | | | | Adjusted residual | | | | | N (%) | | | | Adjusted residual | | | |
| Disagree | | | | 35 (81.4%) | | | | **2.4** | | | | 44 (64.7%) | | | | -0.5 | | | | | 14 (50.0%) | | | | **-2.1** | | | |
| Neither agree nor disagree | | | | 1 (2.3%) | | | | **-2.3** | | | | 9 (13.2%) | | | | 0.6 | | | | | 6 (21.4%) | | | | 1.8 | | | |
| Agree | | | | 7 (16.3%) | | | | -1.0 | | | | 15 (22.1%) | | | | 0.1 | | | | | 8 (28.6%) | | | | 1.0 | | | |
| Missing | | | | 0 (0%) | | | | 0 (0%) | | | | 0 (0%) | | | | 0 (0%) | | | | | 0 (0%) | | | | 0 (0%) | | | |
| **I’d rather answer the questions on paper. (p = 0.002)** | | | | | | | | | | | | | | | | | | | | | | | | | | | | |
|  | | | | 18–39 years old | | | | | | | | 40–59 years old | | | | | | | | | 60+ years old | | | | | | | |
|  | | | | N (%) | | | | Adjusted residual | | | | N (%) | | | | Adjusted residual | | | | | N (%) | | | | Adjusted residual | | | |
| Disagree | | | | 39 (90.7%) | | | | 1.9 | | | | 57 (83.8%) | | | | 0.7 | | | | | 17 (60.7%) | | | | **-3.1** | | | |
| Neither agree nor disagree | | | | 3 (7.0%) | | | | -0.5 | | | | 7 (10.3%) | | | | 0.7 | | | | | 2 (7.1%) | | | | -0.3 | | | |
| Agree | | | | 1 (2.3%) | | | | -1.9 | | | | 3 (4.4%) | | | | **-2.0** | | | | | 9 (32.1%) | | | | **4.6** | | | |
| Missing | | | | 0 (0%) | | | | -0.7 | | | | 1 (1.5%) | | | | 1.0 | | | | | 0 (0%) | | | | -0.5 | | | |
| **I can basically imagine filling out electronic questionnaires. (p = 0.005)** | | | | | | | | | | | | | | | | | | | | | | | | | | | | |
|  | | | | 18–39 years old | | | | | | | | 40–59 years old | | | | | | | | | 60+ years old | | | | | | | |
|  | | | | N (%) | | | | Adjusted residual | | | | N (%) | | | | Adjusted residual | | | | | N (%) | | | | Adjusted residual | | | |
| Disagree | | | | 0 (0%) | | | | **-2.1** | | | | 3 (4.4%) | | | | -1.0 | | | | | 6 (21.4%) | | | | **3.6** | | | |
| Neither agree nor disagree | | | | 4 (9.3%) | | | | 0.4 | | | | 7 (10.3%) | | | | 1.0 | | | | | 0 (0%) | | | | -1,7 | | | |
| Agree | | | | 38 (88.4%) | | | | 0.8 | | | | 58 (85.3%) | | | | 0.1 | | | | | 22 (78.6%) | | | | -1.0 | | | |
| Missing | | | | 1 (2.3%) | | | | 1.5 | | | | 0 (0%) | | | | -1.0 | | | | | 0 (0%) | | | | 0.5 | | | |
| **Completion time in minutes (N = 138, p = 0.002,** ^a^means differ significantly from each other) | | | | | | | | | | | | | | | | | | | | | | | | | | | | |
| 18–39 years old^a^ | | | | | 40–59 years old | | | | | | | | 60+ years old^a^ | | | | | | | | | F(2) | | | | η^2^ | | |
| Mean | | SD | | | Mean | | | | SD | | | | Mean | | | | | SD | | | |  | | | |  | | |
| 6.08 | | 2.08 | | | 6.93 | | | | 2.25 | | | | 8.16 | | | | | 3.10 | | | | 6.288 | | | | 0.085 | | |
| **School diploma (N = 136)** | | | | | | | | | | | | | | | | | | | | | | | | | | | | |
| **By filling in and storing the data on my goals and benefits, the relationship with my physician can be improved. (p = 0.011)** | | | | | | | | | | | | | | | | | | | | | | | | | | | | |
|  | | | | No degree OR lower degree | | | | | | | intermediate secondary degree OR polytechnic high school degree OR university of applied sciences entrance qualification | | | | | | higher education entrance qualification | | | | | | | other degree | | | | |
|  | | | | N (%) | | | Adjusted residual | | | | N (%) | | | Adjusted residual | | | N (%) | | | Adjusted residual | | | | N (%) | | | | Adjusted residual |
| Disagree | | | | 1 (4.0%) | | | -1.7 | | | | 13 (18.8%) | | | 1.4 | | | 5 (13.2%) | | | -0.3 | | | | 1 (25.0%) | | | | 0.6 |
| Neither agree nor disagree | | | | 3 (12%) | | | **-2.2** | | | | 19 (27.5%) | | | -0.7 | | | 18 (47.4%) | | | **2.7** | | | | 1 (30.1%) | | | | -0.2 |
| Agree | | | | 21 (84%) | | | **3.2** | | | | 37 (53.6%) | | | -0.4 | | | 15 (39.5%) | | | **-2.3** | | | | 2 (50.0%) | | | | -0.2 |
| Missing | | | | 0 (0%) | | | / | | | | 0 (0%) | | | / | | | 0 (0%) | | | / | | | | 0 (0%) | | | | / |
| **What is the maximum amount of minutes it may take, that you would fill in the data monthly? (p = 0.043)** | | | | | | | | | | | | | | | | | | | | | | | | | | | | |
|  | | | | No degree OR lower degree | | | | | | | intermediate secondary degree OR polytechnic high school degree OR university of applied sciences entrance qualification | | | | | | higher education entrance qualification | | | | | | | other degree | | | | |
|  | | | | N (%) | | | Adjusted residual | | | | N (%) | | | Adjusted residual | | | N (%) | | | Adjusted residual | | | | N (%) | | | | Adjusted residual |
| That would be too often for me | | | | 0 (0%) | | | -0.5 | | | | 0 (0%) | | | -1.0 | | | 1 (2.6%) | | | 1.6 | | | | 0 (0%) | | | | -0.2 |
| 1–5 minutes | | | | 2 (8.0%) | | | -1,7 | | | | 17 (24.6%) | | | 1.2 | | | 7 (18.4%) | | | -0.4 | | | | 2 (50.0%) | | | | 1.5 |
| 10–20 minutes | | | | 22 (88%) | | | **2.6** | | | | 44 (63.8%) | | | -0.4 | | | 21 (55.3%) | | | -1.6 | | | | 2 (50.0%) | | | | -0.7 |
| 30+ minutes | | | | 1 (4.0%) | | | -1.2 | | | | 5 (7.2%) | | | -1.4 | | | 9 (23.7%) | | | **2.9** | | | | 0 (0%) | | | | -0.7 |
| Missing | | | | 0 (0%) | | | -0.8 | | | | 3 (4.3%) | | | 1.7 | | | 0 (0%) | | | -1.1 | | | | 0 (0%) | | | | -0.3 |
| **How often would you be willing to fill in the questionnaires on your personal goals and benefits? (p = 0.045)** | | | | | | | | | | | | | | | | | | | | | | | | | | | | |
|  | | | | No degree OR lower degree | | | | | | | intermediate secondary degree OR polytechnic high school degree OR university of applied sciences entrance qualification | | | | | | higher education entrance qualification | | | | | | | other degree | | | | |
|  | | | | N (%) | | | Adjusted residual | | | | N (%) | | | Adjusted residual | | | N (%) | | | Adjusted residual | | | | N (%) | | | | Adjusted residual |
| Rarer than at each patient-physician consultation | | | | 5 (20%) | | | -0.4 | | | | 18 (26.1%) | | | 0.9 | | | 8 (21.1%) | | | -0.3 | | | | 0 (0%) | | | | -1.1 |
| At every patient-physician consultation | | | | 16 (64.0%) | | | 0.2 | | | | 41 (59.4%) | | | -0.8 | | | 26 (68.4%) | | | 0.9 | | | | 2 (50.0%) | | | | -0.5 |
| More frequently than at each patient-physician consultation | | | | 0 (0%) | | | -1.9 | | | | 8 (11.6%) | | | 0.5 | | | 4 (10.5%) | | | 0.1 | | | | 2 (50.0%) | | | | **2.7** |
| Missing | | | | 4 (16%) | | | **3.1** | | | | 2 (2.9%) | | | -0.9 | | | 0 (0.0%) | | | -1.6 | | | | 0 (0.0%) | | | | -0.4 |
| **Completion time in minutes (N = 135, p = 0.006,** ^a, b^means with same subscript differ significantly from each other) | | | | | | | | | | | | | | | | | | | | | | | | | | | | |
| No degree OR lower degree^a^ | | | Intermediate secondary degree OR polytechnic high school degree OR university of applied sciences entrance qualification^b^ | | | | | | | Higher education entrance qualification^a, b^ | | | | | Other degree | | | | | | | | F(3) | | | | η^2^ | |
| Mean | SD | | Mean | | | SD | | | | Mean | | | SD | | Mean | | | | SD | | | |  | | | |  | |
| 8.02 | 2.52 | | 7.03 | | | 2.59 | | | | 5.84 | | | 1.91 | | 7.25 | | | | 3.20 | | | | 4.294 | | | | 0.090 | |
| **ePBI version: app vs web (N = 139)** | | | | | | | | | | | | | | | | | | | | | | | | | | | | |
| **The questionnaire on my personal goals and benefits forms a good basis for a patient-physician consultation. (p = 0.027)** | | | | | | | | | | | | | | | | | | | | | | | | | | | | |
|  | | | | Web | | | | | | | | | | | | | App | | | | | | | | | | | |
|  | | | | N (%) | | | | | | | Adjusted residual | | | | | | N (%) | | | | | | | Adjusted residual | | | | |
| Disagree | | | | 3 (4.5%) | | | | | | | -1.6 | | | | | | 9 (12.3%) | | | | | | | 1.6 | | | | |
| Neither agree nor disagree | | | | 9 (13.6%) | | | | | | | -1.8 | | | | | | 19 (26.0%) | | | | | | | 1.8 | | | | |
| Agree | | | | 54 (81.8%) | | | | | | | **2.8** | | | | | | 44 (60.3%) | | | | | | | **-2.8** | | | | |
| Missing | | | | 0 (0%) | | | | | | | -1.0 | | | | | | 0 (0%) | | | | | | | 1.0 | | | | |
